# Free factor XIII-B in atypical hemolytic uremic syndrome: a remission signal

**DOI:** 10.1016/j.rpth.2026.107006

**Published:** 2026-10-09

**Authors:** Arijit Biswas, Sneha Singh, Johannes Oldenburg

**Affiliations:** Institut für Exp. Hämatologie und Transfusionsmedizin (IHT), Universitätsklinikum Bonn, Bonn, Germany

Complement-mediated thrombotic microangiopathy, still widely termed atypical hemolytic uremic syndrome (aHUS), sits at the intersection of complement activation, endothelial injury, coagulation, and fibrinolysis. Yet, its clinical monitoring depends almost entirely on the downstream consequences of microvascular injury: thrombocytopenia, hemolysis, and declining kidney function. By the time these are clear, tissue damage is often established. An early biomarker for thrombotic microangiopathy would be valuable for guiding the duration of individualized complement therapy and monitoring after treatment withdrawal.

In this issue, Janszky et al. [1] carry that search beyond complement by profiling coagulation factor XIII (FXIII) in 102 patients with aHUS and 73 healthy controls, and combining plasma phenotyping with systematic high-resolution melting and Sanger sequencing of the F13A1 and F13B loci. The genetic result is cleanly negative. Six common and 4 rare nonsynonymous coding variants were identified, none enriched relative to European reference populations, and, apart from the known loss-of-function F13B Tyr100Ter allele, genotype did not explain the plasma phenotype. Negative findings of this kind are undervalued; this one narrows the mechanistic field and argues against coding variation in FXIII as an inherited driver of aHUS, with the honest caveat, which the authors state, that a melting-based screen of coding regions and splice sites cannot see deep intronic, regulatory, or structural variation.

The plasma data are rather more provocative. In acute disease, FXIII activity and FXIII-A2B2 were reduced (median activity, 72.8% vs 136% in controls), whereas free FXIII-B was elevated (12.7 vs 9.4 mg/L). In hematologic remission, activity and FXIII-A2B2 returned to control values, yet total and free FXIII-B climbed higher (free FXIII-B, 14.7 mg/L). This acute pattern is biologically plausible: as FXIII-A is activated and incorporated into fibrin-rich microthrombi, the complex is consumed, and B subunits are liberated [2]. Elevated D-dimer levels also support ongoing fibrin turnover, albeit without identifying the specific site. One point in the wording deserves correction because the concluding sentence of the article puts it the other way around: there was no persistent FXIII deficiency in remission. Activity and FXIII-A2B2 were statistically indistinguishable from those of controls, and the case against causality rests on the absence of variant enrichment together with that normalization, not on a residual deficit.

The remission finding is the study’s central observation and its central puzzle. FXIII-B is no inert appendage. It circulates in roughly 2-fold molar excess to FXIII-A, stabilizes FXIII-A reciprocally, and mediates the interaction of the zymogen with fibrinogen [3], [4], [5]. Its 10 sushi domains depend on structural disulfides for secretion, oligomeric state, and, for selected variants, control of FXIII-A activation [6], and proteomic screening places complement C1q and C3, alongside alpha-2-macroglobulin, among candidate partners of the free subunit [7]. None of this establishes a physiological role in complement regulation, and the distinction matters in this disease: despite the sushi-domain architecture it shares with complement factor H, FXIII-B did not behave as a factor H-like regulator when the 2 were compared directly [8]. Transcriptomic analysis, meanwhile, links F13A1 expression to macrophage polarization and inflammation while confirming a hepatic origin for F13B [9]. An expanded free pool in remission may therefore reflect altered synthesis or inflammatory regulation rather than any complement-directed activity of the protein itself.

Whether that pool marks remission is a separate question, and it is not yet settled. Only 4 of 89 patients contributed paired samples, so the acute-to-remission rise is a contrast between groups rather than a trajectory within patients. The patients were also considerably younger than the controls (mean, 23.9 vs 38.4 years), and subunit antigen levels in healthy individuals vary with age and sex [10]; comparing a younger, partly pediatric cohort with older controls is confounded for precisely the analyte at issue. Remission was defined hematologically without requiring renal recovery, and 35% of that group had reached end-stage kidney disease, a state associated with altered FXIII activity and antigen [11] and with inflammation, altered hepatic synthesis, and altered protein turnover, even though renal filtration is unlikely to clear a protein of this size. Free FXIII-B was, moreover, already elevated in acute disease, so it is not remission-specific.

Directionality follows from this. A marker highest in remission may report recovery, a durable footprint of past injury, or ongoing subclinical activity, and these readings are not interchangeable; only the last would support the surveillance role the authors envisage. This interpretation also reverses the expected clinical logic. If free FXIII-B is highest in quiescence and lower in acute disease, then a fall, not a rise, would herald relapse, which is the opposite of what the consumption model predicts—a hypothesis that only serial sampling can test (Figure). Discrimination, meanwhile, remains unaddressed. The acute and remission distributions show substantial overlap; their group-level difference is modest (*P* = .02), and no sensitivity, specificity, or threshold analysis is presented. Group-level separation and individual-level classification are distinct; only the former has been demonstrated.

**Figure:**
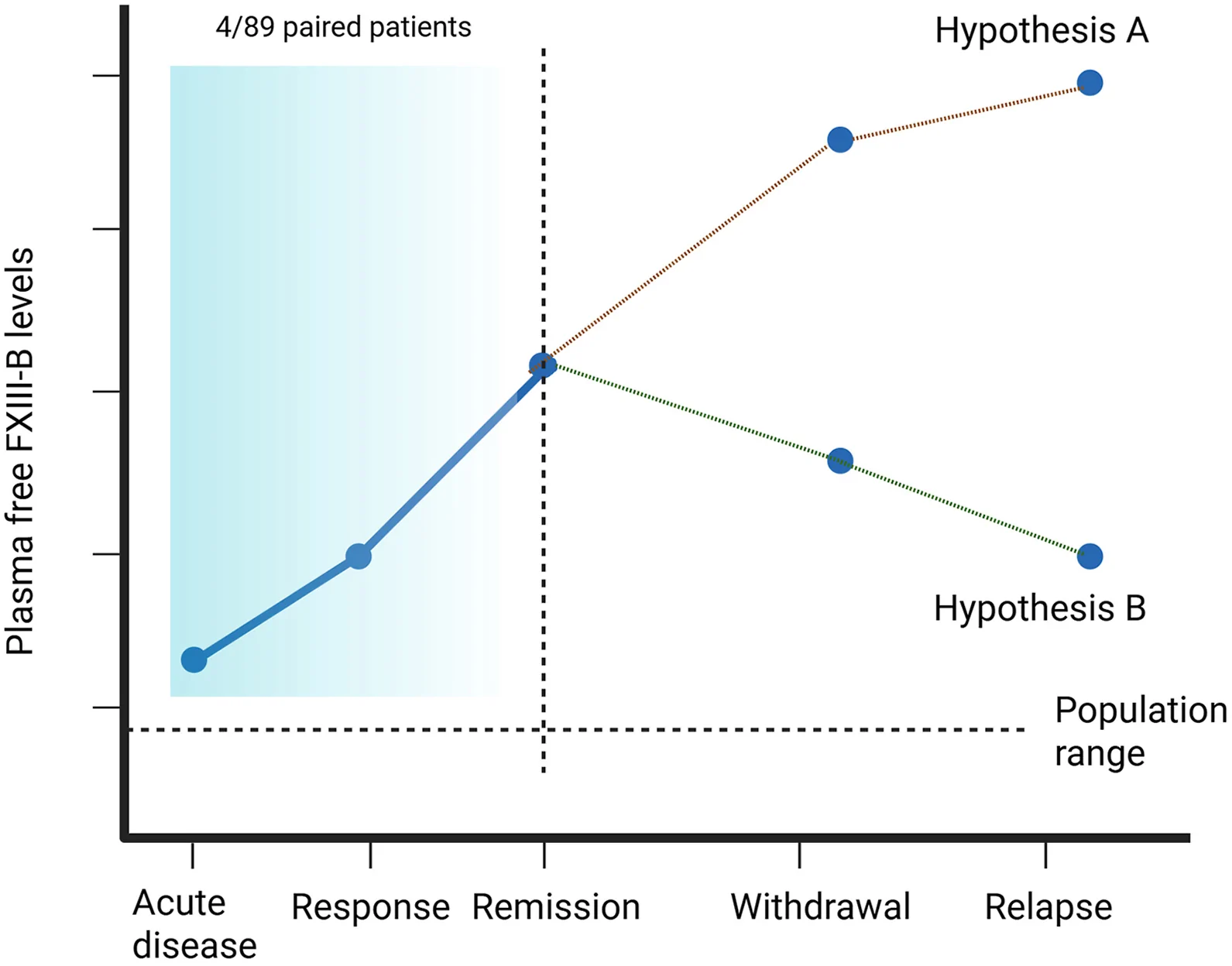
Conceptual free FXIII-B trajectory across the aHUS disease course. The acute-to-remission rise reflects cross-sectional group medians only (4 of 89 patients had paired samples, shown by hatching). Two competing, currently indistinguishable hypotheses for the postremission trajectory are shown: free FXIII-B staying elevated before relapse (consumption model) versus falling before relapse (as the reported cross-sectional pattern would predict). Serial sampling is required to resolve which, if either, holds. aHUS, atypical hemolytic uremic syndrome; FXIII, factor XIII.

The 2 halves of the paper intersect in a way worth drawing out. In the F13B Tyr100Ter family, heterozygous carriers, including the clinically healthy father, had reduced free FXIII-B, the very analyte proposed as a marker, displaced by genotype in the direction opposite to the disease signal [1], [12]. Heterozygous F13B loss-of-function alleles are individually rare but collectively not negligible. Any interpretation based on an absolute free FXIII-B concentration will therefore need to account for F13B genotype, or at minimum total FXIII-B and the free-to-total ratio, as a covariate, which is a further argument for within-patient change over fixed population thresholds.

The decisive study is a longitudinal one. Serial plasma sampling should begin before plasma therapy or complement inhibition where feasible, continue through hematologic response and renal recovery, extend into stable follow-up, and concentrate around treatment withdrawal and relapse, with free and total FXIII-B, FXIII-A_2_B_2_, and FXIII activity measured alongside platelet count, lactate dehydrogenase, haptoglobin, creatinine, proteinuria, D-dimer, and markers of complement activation. Three questions will determine its clinical value: whether within-patient change outperforms between-person concentration; whether free FXIII-B adds discrimination beyond tests already in routine use rather than tracking them; and whether the signal is specific, which requires comparison against other thrombotic microangiopathies, chronic kidney disease and dialysis, systemic inflammation, and non-complement-mediated thrombotic states. A signal tied broadly to fibrin turnover would still be informative, but it would be a different clinical instrument.

Analytical readiness is the quieter requirement. The immunoassays used here are well characterized, and the coherence across the FXIII measurements is reassuring, but a deployable test needs published imprecision, freeze-thaw and storage stability, reference intervals, interlaboratory reproducibility, and demonstrated freedom from interference by abundant plasma proteins, particularly because the proposed application depends on detecting modest longitudinal shifts rather than gross deficiency.

Clinical stakes: Relapse risk following eculizumab discontinuation is heterogeneous and driven by underlying complement genetics; dynamic monitoring tools remain a critical unmet need [13].

Current translation: Free FXIII-B measurement should not currently guide decisions to initiate, withhold, or resume complement inhibition.

Future utility: If longitudinal validation confirms its predictive power, free FXIII-B could fit into a broader panel tracking genetic risk, complement activation, endothelial injury, and fibrin turnover—an approach far more viable than relying on a single surrogate analyte.

Core takeaway: Janszky et al. provide evidence against F13A1 and F13B coding variation as an inherited driver of aHUS, while revealing that remission retains a measurable hemostatic signature (Table). Validating whether serial monitoring of this signature predicts clinical relapse is the essential work ahead.

**Table:** Free FXIII-B as a candidate biomarker in atypical hemolytic uremic syndrome: what the present study establishes and what validation still requires.

Criterion	Evidence provided by Janszky et al.	Requirement before clinical use
Association with disease state	Free FXIII-B higher in acute aHUS and higher again in remission versus controls	Replication in an independent, contemporaneously sampled cohort
Temporal direction	Cross-sectional group contrast; only 4 of 89 patients had paired acute and remission samples	Within-patient trajectories spanning acute disease, response, remission, withdrawal, and relapse. Evaluate within-patient changes in free FXIII-B over time rather than compare values with population reference ranges
Discrimination	Group medians differ; distributions of acute and remission samples overlap; no threshold analysis	Sensitivity, specificity, and decision thresholds for the intended clinical question
Confounding	Patients younger than controls; 35% of remission group with ESKD; treatment exposure not modeled	Adjustment for age, sex, renal function, dialysis, inflammation, and complement-directed therapy
Genetic modification of the analyte	Heterozygous *F13B* Tyr100Ter carriers, including an unaffected relative, had reduced free FXIII-B	*F13B* genotype, total FXIII-B, and the free-to-total ratio treated as covariates
Specificity	Not assessed outside aHUS	Comparison with other TMAs, chronic kidney disease, dialysis, inflammation, and other thrombotic states
Analytical performance	Well-characterized research immunoassays	Imprecision, stability, reference intervals, interlaboratory reproducibility, interference testing

aHUS, atypical hemolytic uremic syndrome; ESKD, end-stage kidney disease; FXIII, factor XIII; TMA, thrombotic microangiopathy.
